# Association of Sleep Patterns and Sleep Quality with Academic Performance Among Female University Students: Insights Supporting SDG 3 (Good Health and Well-Being)

**DOI:** 10.3390/clockssleep8030039

**Published:** 2026-06-29

**Authors:** Noorah Saleh Al-Sowayan, Lama Essam Aboselmiya

**Affiliations:** Department of Biology, College of Science, Qassim University, Buraydah 52571, Saudi Arabia

**Keywords:** sleep quality, academic performance, PSQI, university students, sleep patterns, cognitive health

## Abstract

Background: Sleep quality plays an essential role in cognitive performance, memory consolidation, learning efficiency, and overall wellbeing. University students are particularly vulnerable to sleep disturbances because of academic stress, irregular sleep schedules, and lifestyle-related factors. Poor sleep quality has been associated with impaired academic performance and reduced cognitive functioning. Objective: This study aimed to evaluate the relationship between sleep patterns, sleep quality, and academic performance among female university students. Methods: A cross-sectional study was conducted among 201 female university students at Qassim University, Saudi Arabia. Data were collected using an electronic self-administered questionnaire that included demographic characteristics, sleep-related behaviors, and the Pittsburgh Sleep Quality Index (PSQI). Academic performance was assessed using self-reported Grade Point Average (GPA). Statistical analysis included descriptive statistics, Spearman correlation analysis, one-way ANOVA with Tukey post hoc analysis, Chi-square tests, and multiple linear regression analysis. Results: The mean GPA of the participants was 4.13 ± 0.60 (on a 5-point scale), while the mean PSQI score was 8.81 ± 3.26, indicating generally poor sleep quality. A significant negative correlation was observed between PSQI score and GPA (rho = −0.200, *p* = 0.0047). Students with good sleep quality demonstrated significantly higher GPA scores compared with students with poor sleep quality (F(2,194) = 6.31, *p* = 0.0022). Significant associations were also identified between sleep quality and both bedtime (*p* = 0.0009) and sleep duration category (*p* = 0.0002). However, after adjustment for other variables, the independent effect of PSQI on GPA was attenuated and did not reach statistical significance (*p* = 0.138). This discrepancy between the significant bivariate correlation (rho = −0.200, *p* = 0.005) and the non-significant multivariate result represents the most important finding of this study, suggesting that sleep quality alone does not independently predict GPA when other academic and behavioral factors are considered. Conclusion: Poor sleep quality was highly prevalent among female university students and showed a significant bivariate association with lower academic performance, though this relationship was attenuated in the multivariate model. Promoting healthy sleep behaviors may support student wellbeing and academic functioning, cognitive wellbeing, quality of life, and the advancement of Sustainable Development Goal 3 (Good Health and Well-Being).

## 1. Introduction

Sleep is a fundamental physiological process essential for maintaining human health, cognitive performance, emotional regulation, metabolic balance, and overall wellbeing. Adequate sleep is necessary for optimal brain functioning and plays a central role in attention, memory consolidation, learning efficiency, decision-making, and executive functioning. In contrast, sleep deprivation and poor sleep quality have been associated with impaired concentration, reduced productivity, mood disturbances, weakened immune responses, increased psychological stress, and deterioration in quality of life [1,2].

Recent evidence further supports the critical role of sleep in memory consolidation and next-day learning capacity. Systematic reviews have confirmed that sleep benefits memory across multiple life stages, with both REM and NREM stages contributing to neuronal recovery and synaptic plasticity [3,4]. Guttesen et al. demonstrated that overnight memory consolidation directly supports next-day learning by facilitating hippocampal-to-neocortical memory transfer [5].

Sleep is regulated through highly complex neurophysiological mechanisms involving circadian rhythm synchronization, hormonal regulation, and neural restorative processes. Both rapid eye movement (REM) sleep and non-rapid eye movement (NREM) sleep contribute significantly to neuronal recovery, synaptic plasticity, emotional processing, and memory consolidation [6,7]. The disturbance of these physiological sleep cycles may negatively affect higher cognitive functions, including attention span, information processing speed, working memory, and learning performance [6,7].

The global prevalence of sleep disturbances among university students is substantial. A recent systematic review and meta-analysis of 95,938 undergraduate students reported a pooled prevalence of insomnia symptoms of 46.9%, highlighting the scale of this public health challenge [8]. Gardani et al. similarly demonstrated high rates of poor sleep and psychological stress among undergraduate students across multiple countries [9].

Globally, sleep disturbances among university students have become an increasing public health concern. University students represent a particularly vulnerable population because of academic demands, examination stress, prolonged electronic device use, irregular sleep schedules, social activities, and unhealthy lifestyle behaviors [2,10]. Several studies have reported high rates of insufficient sleep duration, delayed sleep timing, excessive daytime sleepiness, and poor sleep quality among university students across different countries and educational settings [2,10].

Psychological interventions targeting sleep quality in university students have also demonstrated efficacy, with cognitive behavioral therapy for insomnia (CBT-I) showing the strongest effect sizes, underscoring the importance of university-level sleep health programs [11].

In Saudi Arabia, student wellbeing and healthy lifestyle promotion have become important priorities within Saudi Vision 2030 and national quality-of-life initiatives. Increasing attention has been directed toward preventive healthcare, mental wellbeing, cognitive performance, and health promotion among young adults and university students [1,2]. Improving sleep health is also closely aligned with the United Nations Sustainable Development Goal 3 (Good Health and Well-Being), which emphasizes promoting healthy lives, mental wellbeing, disease prevention, and improved quality of life across all age groups [1]. Sleep quality has therefore become increasingly connected to preventive medicine, university wellbeing programs, and national research priorities focusing on mental health and lifestyle-related health behaviors [1,2].

Accumulating evidence has demonstrated that chronic sleep deprivation negatively affects multiple cognitive domains, including attention, executive function, information processing speed, working memory, and academic learning ability [6,7]. Sleep is considered a critical component of memory consolidation since it facilitates neural restoration processes that occur during both REM and NREM sleep stages. Experimental and clinical studies have shown that disrupted sleep architecture may impair long-term memory retention, reduce cognitive flexibility, and negatively affect academic performance [6,7].

Systematic reviews examining sleep and academic performance in university students have identified sleep quality and daytime dysfunction as the parameters most consistently associated with GPA [12,13]. Notably, Suardiaz-Muro et al. demonstrated that poor sleep quality, sleep irregularity, and delayed bedtime were all negatively associated with academic performance, with daytime dysfunction emerging as a particularly robust predictor [12]. These findings suggest that analyzing individual PSQI domains, rather than relying solely on the global score, may yield more targeted insights for intervention.

The Pittsburgh Sleep Quality Index (PSQI), developed by Buysse et al., is among the most widely used standardized instruments for evaluating sleep quality in both clinical and research settings [14]. The PSQI assesses several important dimensions of sleep, including subjective sleep quality, sleep latency, sleep duration, sleep efficiency, sleep disturbances, use of sleep medication, and daytime dysfunction [14]. Because of its validity and reliability, the PSQI has been extensively applied in studies evaluating sleep quality among university students and populations exposed to psychological or cognitive stress [2,14].

Chronotype—an individual’s biological preference for sleep and wake timing—represents an important but frequently overlooked determinant of sleep quality and academic performance. Evening-type individuals are particularly susceptible to social jet lag, defined as the misalignment between biological and social clock schedules, which has been independently associated with poorer cognitive performance and lower academic achievement [15,16]. Figueiredo and Kulari demonstrated that evening chronotype and late-night sleep preferences negatively impacted academic performance among university students, independent of sleep duration [17]. Alshahrani et al. similarly found that evening-type students in a university setting showed associations with unhealthy lifestyle behaviors and academic difficulties, underscoring the importance of chronotype assessment in sleep research [18]. The present study did not assess chronotype, which represents an important limitation acknowledged in the limitations section.

Emerging evidence suggests that chronic sleep disturbances may contribute to neurodegenerative processes, including impaired glymphatic clearance and amyloid-beta accumulation, which have been linked to long-term cognitive decline [19,20]. Although these mechanisms primarily affect older adults, promoting healthy sleep habits during early adulthood may represent an important preventive health investment [20].

Among university students, inadequate sleep has consistently been associated with reduced academic performance and lower grade point average (GPA). Students experiencing poor sleep quality often report daytime fatigue, impaired concentration, reduced motivation, slower cognitive processing, impaired decision-making ability, and decreased learning capacity, all of which may negatively influence academic achievement [2,6,10,21]. Several international and regional studies, including studies conducted in Saudi Arabia, have demonstrated significant associations between poor sleep quality and lower academic performance among university students [2,10,21].

Within the Saudi Arabian context, Alomri and Alghamdi reported that 78.4% of university students experienced insomnia and 56.6% reported excessive daytime sleepiness, reflecting the high burden of sleep disorders in this population [22]. Alhusseini et al. further demonstrated significant associations between poor sleep quality, academic performance, and psychological distress among medical students at a Saudi university [23]. Notably, Alghamdi et al. reported that no significant association was observed between sleep quality and GPA among clinical-year medical students at King Abdulaziz University, highlighting the inconsistency of findings across regional studies and the influence of contextual factors such as major and academic level [24].

Despite the growing international literature examining sleep quality and academic performance, limited local evidence remains available among female university students in Saudi Arabia. Furthermore, few regional studies have comprehensively evaluated sleep quality using standardized measures such as the Pittsburgh Sleep Quality Index (PSQI) while simultaneously examining sleep duration, bedtime patterns, and academic performance within the broader framework of student wellbeing, preventive health, and quality-of-life initiatives [2,14]. This represents an important research gap that warrants further investigation.

Therefore, the present study aimed to evaluate the relationship between sleep patterns, sleep quality, and academic performance among female university students using the Pittsburgh Sleep Quality Index (PSQI).

Research Question: Is there a significant relationship between sleep quality, sleep patterns, and academic performance among female university students?

Research Hypothesis: Female university students with poorer sleep quality will demonstrate lower academic performance compared with students who report better sleep quality.

General Objective: To evaluate the relationship between sleep quality, sleep patterns, and academic performance among female university students.

Specific Objectives: (1). To assess sleep quality among female university students using the Pittsburgh Sleep Quality Index (PSQI), (2). To examine the relationship between PSQI score and academic performance (GPA), (3). To compare GPA across different sleep quality groups, (4). To investigate the association between sleep quality and sleep-related behaviors such as sleep duration, bedtime, and nap status and (5). To explore whether sleep quality remains associated with academic performance after adjusting for selected demographic and lifestyle variables.

## 2. Results

### 2.1. Participant Characteristics

A total of 201 female university students participated in this study. As presented in Table 1, most participants were aged between 20 and 22 years (64.2%), followed by students older than 22 years (21.4%). Biology students represented the largest proportion of the sample (54.2%), while Mathematics students accounted for 13.4% of the participants. The eighth academic level was the most represented group (26.4%).

### 2.2. Sleep Pattern Characteristics

The sleep-related characteristics of the participants are summarized in Table 2. Most students reported sleeping between 5 and 6 h per night (42.3%), while 29.9% reported sleeping 6–7 h. More than half of the participants (53.7%) reported going to bed after midnight. Additionally, 36.3% of the students reported taking daytime naps.

### 2.3. Overview of GPA, Sleep Quality, and Sleep Duration

As shown in Table 3, the mean GPA of the participants was 4.13 ± 0.60. The mean PSQI score was 8.81 ± 3.26, suggesting generally poor sleep quality among the participants. The average sleep duration was 6.66 ± 2.28 h.

The mean PSQI score exceeded the commonly accepted threshold for poor sleep quality, indicating a high prevalence of impaired sleep among the participants.

### 2.4. Spearman Correlation Analysis Between PSQI Score and GPA

**Figure 1:**
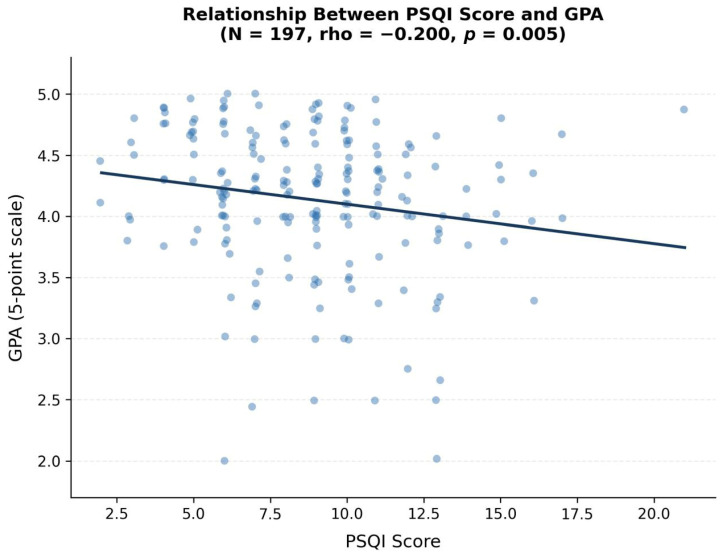
Correlation between PSQI score and GPA.

Spearman correlation analysis demonstrated a significant negative correlation between PSQI score and GPA, as shown in Table 4 (rho = −0.200, *p* = 0.0047). This finding indicates that poorer sleep quality was associated with lower academic performance. This negative relationship is illustrated in Figure 1.

The scatter plot demonstrates a negative association between PSQI score and GPA, showing that increasing sleep disturbance severity was associated with declining academic performance.

### 2.5. GPA Differences Across Sleep Quality Groups

As presented in Table 5, students with good sleep quality had the highest mean GPA (4.46 ± 0.38), whereas students with poor sleep quality had the lowest GPA (3.97 ± 0.64). One-way ANOVA revealed significant differences in GPA among the sleep quality groups (F(2,194) = 6.31, *p* = 0.0022).

**Figure 2:**
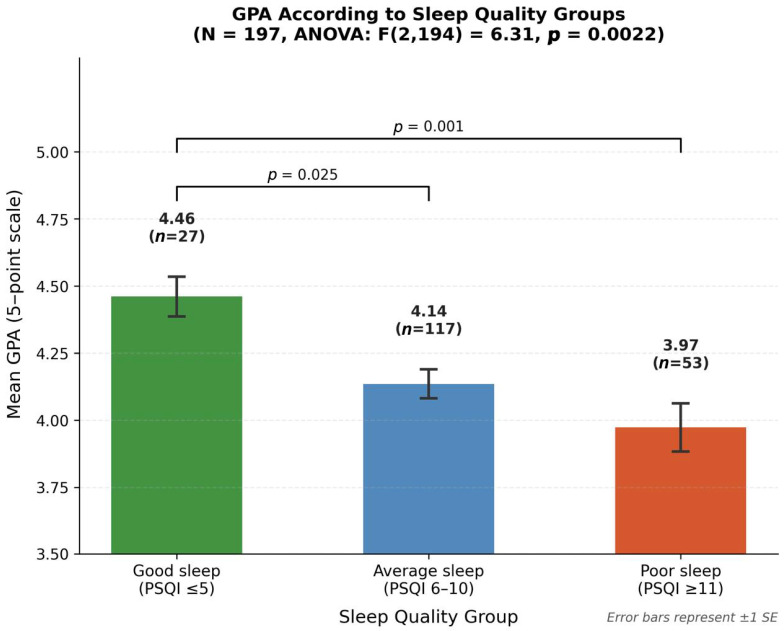
GPA according to sleep quality groups.

Post hoc Tukey analysis demonstrated significant differences between the good sleep group and both the average sleep group (*p* = 0.025) and the poor sleep group (*p* = 0.001). However, no significant difference was observed between the average and poor sleep groups (*p* = 0.213). The differences in GPA across sleep quality groups are illustrated in Figure 2.

Students with good sleep quality demonstrated higher GPA scores compared with students with average or poor sleep quality.

### 2.6. GPA According to Sleep Duration, Bedtime, Academic Level, and Major

As presented in Table 6, no statistically significant differences in GPA were observed according to sleep duration category (F(3,197) = 0.792, *p* = 0.500), bedtime (F(2,198) = 1.896, *p* = 0.153), or academic level (F(7,193) = 0.489, *p* = 0.842).

However, GPA differed significantly according to major (F(7,193) = 2.434, *p* = 0.0206). This finding should be interpreted cautiously because some majors had relatively small sample sizes.

### 2.7. Chi-Square Analysis of Sleep Quality and Categorical Variables

Chi-square analysis revealed significant associations between sleep quality and both bedtime (χ^2^(4) = 18.64, *p* = 0.0009) and sleep duration category (χ^2^(6) = 26.50, *p* = 0.0002), as presented in Table 7. Students who reported going to bed after midnight were more likely to experience poor sleep quality.

No significant associations were found between sleep quality and nap status (*p* = 0.765), academic level (*p* = 0.667), or major (*p* = 0.277).

### 2.8. Independent Predictors of GPA: Multiple Linear Regression Results

Multiple linear regression analysis was conducted to determine whether sleep quality independently predicted GPA after adjusting for sleep duration, academic level, nap status, and major.

As shown in Table 8, the overall regression model was statistically significant (F(6,190) = 2.629, *p* = 0.018), explaining approximately 7.7% of the variance in GPA (R^2^ = 0.077, Adjusted R^2^ = 0.048).

Although PSQI score showed a negative association with GPA (B = −0.021), the relationship did not remain statistically significant after adjustment (*p* = 0.138).

These findings suggest that academic performance is influenced by multiple interacting behavioral and educational factors in addition to sleep quality alone.

## 3. Discussion

The present study investigated the relationship between sleep patterns, sleep quality, and academic performance among female university students. The findings demonstrated that sleep disturbances were common among the participants and were significantly associated with lower academic performance. Students with healthier sleep patterns achieved significantly higher GPA scores compared with students reporting impaired sleep quality.

The mean PSQI score observed in the current study suggested an overall tendency toward inadequate sleep among the participants. This finding is consistent with previous international and regional studies reporting high rates of sleep disturbances among university students due to academic stress, excessive screen exposure, irregular schedules, and lifestyle-related factors [2,10,21]. University students represent a population particularly vulnerable to sleep disruption because of demanding academic environments and altered daily routines.

The present study also demonstrated a significant negative correlation between PSQI score and GPA, indicating that worsening sleep quality was associated with declining academic performance. This finding agrees with previous studies showing that insufficient sleep negatively affects concentration, attention, executive functioning, and learning efficiency [2,6,10]. Sleep is essential for memory consolidation and neural restoration processes, particularly during REM and non-REM sleep stages, which are critical for cognitive performance and academic achievement [6,7].

Students with good sleep quality showed significantly higher GPA scores compared with students experiencing impaired sleep quality. This finding supports the hypothesis that healthy sleep behavior contributes to improved academic outcomes. Similar observations have been reported among university students in different countries, including Saudi Arabia [2,10,21]. Sleep disturbances may impair cognitive processing speed, daytime alertness, information retention, and decision-making ability, ultimately reducing academic productivity and performance [6].

The current study further identified significant associations between sleep quality and both bedtime and sleep duration category. Students who reported sleeping after midnight were more likely to experience poor sleep quality. This observation reflects the growing prevalence of delayed sleep schedules among university students, which may be influenced by academic demands, electronic device use, social media exposure, and unhealthy lifestyle habits [2,10]. Poor sleep hygiene practices may further aggravate these disturbances among university students. Late sleep timing and insufficient sleep duration may disrupt circadian rhythm regulation and contribute to circadian misalignment, negatively affecting cognitive and psychological wellbeing [1,2].

Although the overall regression model was statistically significant, the independent contribution of PSQI to GPA was attenuated and did not remain statistically significant after adjustment for other variables (*p* = 0.138). This finding suggests that academic performance is multifactorial and may also be influenced by additional behavioral, psychological, environmental, and academic factors beyond sleep alone. Nevertheless, the overall regression model remained statistically significant, supporting the role of sleep as an important contributor to academic functioning.

The attenuation of PSQI significance in the multivariate model is consistent with findings from other regional studies. Alghamdi et al. similarly reported no significant association between PSQI score and GPA after adjustment for covariates among Saudi medical students, suggesting that academic performance is multifactorial and that sleep quality may not operate as an independent predictor when other behavioral and educational variables are considered [24]. This observation aligns with the broader literature indicating that the regression model explained 7.7% of GPA variance (R^2^ = 0.077), indicating that unmeasured variables—including chronotype, psychological stress, caffeine intake, screen time, and physical activity—likely account for a substantial portion of the unexplained variance [15,17,25]. Future studies should incorporate these covariates alongside objective sleep measurement and chronotype assessment to better delineate the independent contribution of sleep quality to academic outcomes.

Beyond academic performance, chronic sleep disturbances have been linked to long-term neurological health concerns [19,20,26]. Promoting healthy sleep habits during early adulthood may therefore represent a preventive health investment [26].

The findings of the current study are also relevant to public health priorities, quality-of-life initiatives, and the United Nations Sustainable Development Goal 3, which emphasizes promoting health and wellbeing across all age groups [1]. Within Saudi Arabia, improving student wellbeing and encouraging healthy lifestyle behaviors align with Vision 2030 priorities related to preventive healthcare, mental wellbeing, and quality of life.

Several strengths should be acknowledged in the present study. The study used the Pittsburgh Sleep Quality Index (PSQI), one of the most widely validated instruments for assessing sleep quality [14]. In addition, the study evaluated multiple sleep-related variables, including sleep duration, bedtime, and nap status, while examining their relationship with academic performance.

However, several limitations should also be considered. First, the cross-sectional design limits the ability to establish causal relationships. Second, GPA was self-reported and may therefore be subject to reporting bias. Third, the study included only female students from a single university, which may limit the generalizability of the findings. Finally, some potentially influential variables, such as stress level, caffeine intake, psychological status, physical activity, and screen time, were not evaluated. Fourth, chronotype was not assessed; given that evening chronotype is independently associated with social jet lag and poorer academic outcomes [15,16], its omission may have confounded the relationship between sleep quality and GPA. Fifth, the exclusive use of self-reported sleep measures, including the PSQI, introduces potential recall and social desirability bias, as subjective sleep duration has been shown to differ substantially from objective actigraphy-based measures [13,25]. Sixth, convenience sampling from a single institution limits external validity [8].

Future studies should include larger and more diverse student populations from multiple universities and should investigate additional behavioral and psychological factors affecting sleep quality and academic performance. Longitudinal studies may also provide better understanding of the long-term effects of chronic sleep disturbances on cognitive health, academic achievement, and neurological wellbeing.

## 4. Materials and Methods

### 4.1. Study Design

This study employed a cross-sectional design to investigate the relationship between sleep patterns, sleep quality, and academic performance among female university students. The study aimed to evaluate the association between subjective sleep quality and academic achievement using a standardized sleep assessment instrument.

### 4.2. Study Setting and Participants

The study was conducted among female university students enrolled at Qassim University, Saudi Arabia. Data collection was carried out using an electronic self-administered questionnaire distributed through online communication platforms and student groups during the study period.

A total of 201 female students voluntarily participated in the study. Participants from different academic levels and specialties were included to provide broader representation of university students and to improve variability in sleep-related and academic characteristics. Because the study used voluntary online convenience sampling, the final sample size was determined by the number of eligible complete responses received during the data collection period. This sample size was considered adequate for the planned exploratory correlation, group comparison, and regression analyses. No formal a priori power analysis was conducted; however, a sample of 201 is generally considered sufficient to detect small-to-moderate effect sizes in Spearman correlation and multiple regression analyses at α = 0.05. Regarding GPA comparability across majors: GPA at Qassim University is calculated on a unified 5-point scale applied uniformly across all colleges and departments; no additional standardization was applied, as the grading system is institution-wide and consistent. Bedtime was examined in bivariate analysis (Chi-square) in relation to sleep quality but was not included in the multiple regression model because it showed no direct significant association with GPA (rho = 0.015, *p* = 0.829). The regression model was designed to identify independent predictors of GPA; variables that are not directly associated with the outcome were excluded to maintain parsimony and avoid unnecessary inflation of the model.

### 4.3. Inclusion and Exclusion Criteria

Inclusion Criteria

- Female university students;
- Currently enrolled at the university during the study period;
- Willing to participate voluntarily;
- Able to complete the electronic questionnaire;
- Completed the questionnaire successfully.

Exclusion Criteria

- Incomplete questionnaire responses;
- Duplicate responses;
- Responses containing missing essential variables related to sleep quality or GPA;
- Responses with invalid or non-numerical GPA entries that could not be corrected during data cleaning.

### 4.4. Data Collection Tool

Data were collected using a structured electronic questionnaire developed through Google Forms. The questionnaire was distributed electronically and designed to evaluate demographic characteristics, sleep-related behaviors, sleep quality, and academic performance.

The questionnaire consisted of three main sections:

1. Demographic Characteristics

This section included:

- Age;
- Academic level;
- Major or specialty.

2. Sleep Pattern Assessment

This section included questions related to:

- Average sleep duration;
- Bedtime;
- Wake-up time;
- Daytime nap status.

Participants were asked to report their habitual sleep behaviors and usual sleep schedules during the previous month.

3. Sleep Quality Assessment

Sleep quality was assessed using the Pittsburgh Sleep Quality Index (PSQI), a validated and widely used standardized instrument developed by Buysse et al. [14].

The PSQI evaluates sleep quality during the previous month and consists of seven components:

1. Subjective sleep quality;
2. Sleep latency;
3. Sleep duration;
4. Habitual sleep efficiency;
5. Sleep disturbances;
6. Use of sleep medication;
7. Daytime dysfunction.

Each component is scored from 0 to 3, generating a total score ranging from 0 to 21. Higher PSQI scores indicate poorer sleep quality.

The PSQI is widely recognized as a reliable instrument for assessing subjective sleep quality in both clinical and research settings and has been extensively applied in studies involving university students and cognitively stressed populations [2,14].

For analytical purposes, PSQI scores were further categorized into good, average, and poor sleep quality groups to facilitate comparative statistical analysis among participants. Specifically: good sleep quality (PSQI ≤ 5), average sleep quality (PSQI 6–10), and poor sleep quality (PSQI ≥ 11). A global PSQI score above 5 is widely used to indicate clinically significant poor sleep quality [14].

#### 4.4.1. Academic Performance Assessment

Academic performance was evaluated using self-reported cumulative Grade Point Average (GPA). Participants entered their cumulative GPA through the electronic questionnaire. GPA values were subsequently reviewed during data cleaning to identify invalid or incorrectly formatted entries before statistical analysis.

#### 4.4.2. Data Management and Cleaning

Collected data were exported into Microsoft Excel for cleaning, coding, and preparation prior to statistical analysis. Data cleaning procedures included:

- Removal of duplicate responses;
- Standardization of major names and specialty labels;
- Correction and conversion of GPA entries into valid numerical values;
- Coding of sleep-related variables;
- Identification and exclusion of missing values where applicable;
- Calculation of total PSQI scores.

The cleaned dataset was reviewed prior to statistical analysis to ensure consistency and validity of the entered responses.

### 4.5. Statistical Analysis

Data were cleaned prior to analysis, including standardization of major names, correction of GPA entries into valid numerical values, and coding of sleep-related variables. Missing values were excluded from variable-specific analyses.

The total Pittsburgh Sleep Quality Index (PSQI) score was calculated for each participant. For analytical purposes, sleep quality was further categorized into good, average, and poor sleep groups.

Continuous variables were expressed as mean ± standard deviation (SD), whereas categorical variables were presented as frequencies and percentages.

Data normality was assessed using the Shapiro–Wilk test. Since several variables were not normally distributed, Spearman correlation analysis was used to examine the relationship between PSQI scores and GPA.

One-way analysis of variance (ANOVA) was conducted to compare GPA across sleep quality groups, followed by Tukey post hoc analysis for pairwise comparisons.

Chi-square tests were used to assess associations between sleep quality and categorical variables, including bedtime, sleep duration category, nap status, academic level, and major. Results involving variables with small subgroup counts were interpreted cautiously.

Multiple linear regression analysis was performed to evaluate whether sleep quality independently predicted GPA after adjusting for sleep duration, nap status, academic level, and major. Academic major was self-reported and categorized into three groups: Sciences (Biology, Chemistry, Physics, Mathematics, Statistics, Health Sciences, Computer Science; *n* = 180), Humanities (English/Translation, Arabic Language, Islamic Studies; *n* = 8), and Other (Administrative Sciences, IT Management, Design, Engineering; *n* = 13), with Sciences as the reference category. Academic level refers to the semester of enrollment, ranging from Level 1 (first semester) to Level 8 (final semester) within the undergraduate program at Qassim University.

Statistical significance was considered at *p* < 0.05.

### 4.6. Ethical Considerations

Participation in this study was voluntary and anonymous. No personal identifiers, biological samples, clinical procedures, or sensitive personal information were collected. Electronic informed consent was obtained from all participants prior to participation. Data were collected through an anonymous online questionnaire and were used solely for research purposes.

## 5. Conclusions

The present study demonstrated that impaired sleep quality was highly prevalent among female university students and showed a significant negative bivariate association with GPA (rho = −0.200, *p* = 0.005). However, this association was attenuated in the multivariate model, indicating a weak and multifactorial relationship.

These findings highlight the importance of promoting healthy sleep behaviors among university students as part of preventive health, student wellbeing, and academic support strategies. Healthy sleep behaviors remain an important component of student wellbeing and academic functioning.

Universities and healthcare institutions should consider implementing sleep awareness programs, mental wellbeing initiatives, and healthy lifestyle interventions aimed at improving sleep hygiene among students. Further research is recommended to investigate additional factors influencing sleep quality and to explore the long-term neurological and academic consequences of chronic sleep disturbances. These findings support the importance of integrating sleep health promotion into university wellbeing initiatives and public health strategies aligned with SDG 3 and Saudi Vision 2030. Early promotion of healthy sleep behaviors during young adulthood may represent an important long-term investment in cognitive health, neurological wellbeing, and academic success.

## Figures and Tables

**Table 1:** Demographic characteristics of the participants (N = 201).

Variable	Category	N	%
Age	<20 years	29	14.4
Age	20–22 years	129	64.2
Age	>22 years	43	21.4
Academic level	First	4	2.0
Academic level	Second	18	9.0
Academic level	Third	16	8.0
Academic level	Fourth	26	12.9
Academic level	Fifth	39	19.4
Academic level	Sixth	27	13.4
Academic level	Seventh	18	9.0
Academic level	Eighth	53	26.4

**Table 2:** Sleep pattern characteristics of the participants.

Variable	Category	N	%
Sleep duration	<5 h	22	10.9
Sleep duration	5–6 h	85	42.3
Sleep duration	6–7 h	60	29.9
Sleep duration	>7 h	34	16.9
Bedtime	Before 10 p.m.	12	6.0
Bedtime	10 p.m.–12 a.m.	81	40.3
Bedtime	After 12 a.m.	108	53.7
Nap status	Yes	73	36.3
Nap status	No	128	63.7

**Table 3:** Descriptive statistics of GPA, PSQI, and sleep duration.

Variable	N	Mean ± SD
GPA	201	4.13 ± 0.60
PSQI score	197	8.81 ± 3.26
Sleep duration (hours)	200	6.66 ± 2.28

Missing values were excluded from variable-specific analyses.

**Table 4:** Correlation between PSQI score and GPA.

Variables	Correlation Coefficient (rho)	*p*-Value
PSQI score vs. GPA	−0.200	0.0047

**Table 5:** Comparison of GPA according to sleep quality groups.

Sleep Quality Group	N	Mean GPA ± SD
Good sleep	27	4.46 ± 0.38
Average sleep	117	4.14 ± 0.59
Poor sleep	53	3.97 ± 0.64

ANOVA: F(2,194) = 6.31, *p* = 0.0022.

**Table 6:** Comparison of GPA according to sleep duration, bedtime, academic level, and major.

Variable	Test Statistic	*p*-Value
Sleep duration category	F(3,197) = 0.792	0.500
Bedtime	F(2,198) = 1.896	0.153
Academic level	F(7,193) = 0.489	0.842
Major	F(7,193) = 2.434	0.0206

**Table 7:** Association between sleep quality and categorical variables.

Variable	Test	*p*-Value
Bedtime	Chi-square	0.0009
Sleep duration category	Chi-square	0.0002
Nap status	Chi-square	0.765
Academic level	Chi-square	0.667
Major	Chi-square	0.277

**Table 8:** Multiple linear regression analysis predicting GPA.

Variable	B	SE	95% CI	*p*-Value
PSQI Score	−0.021	0.014	[−0.049, 0.007]	0.138
Sleep Duration (h)	0.024	0.021	[−0.017, 0.064]	0.245
Academic Level (1–8)	0.021	0.021	[−0.019, 0.062]	0.302
Nap Status (1 = Yes)	−0.025	0.088	[−0.198, 0.149]	0.781

Major was included in the regression model as a categorical covariate but is not displayed in the table for brevity. Model statistics: F(6,190) = 2.629, *p* = 0.018, R^2^ = 0.077, Adj.R^2^ = 0.048. Major: Sciences (ref, *n* = 180), Humanities (*n* = 8), Other (*n* = 13). Other vs. Sciences: B = 0.454, *p* = 0.008. GPA on 5-point scale.

## Data Availability

The original contributions presented in this study are included in the article. Further inquiries can be directed to the corresponding author.
